# Interdisciplinary and integrated clinical management of complex dental disorders

**DOI:** 10.6026/973206300212075

**Published:** 2025-07-31

**Authors:** Neha Choudhary, Anand M Hiremath, Mahesh P.C, Deepa Bullappa, Vinod Kumar M., Ananthalekshmy Rajeev

**Affiliations:** 1 Department of Orthodontics & Dentofacial Orthopaedic, Private Practitioner, Dantalaya Care Dental Clinic, Patna, Bihar, India; 2 Department of Public Health Dentistry, Maratha Mandal Nathajirao G. Halgekar Institute of Dental Sciences, Belagavi, Karnataka, India; 3 Department of Public Health Dentistry, The Oxford Dental College, Bangalore, Karnataka, India

**Keywords:** Interdisciplinary, dental disorders

## Abstract

This prospective cohort study compare interdisciplinary versus conventional single-specialty approaches in managing complex dental conditions among 240 patients. The interdisciplinary group showed significantly higher treatment success (92.5% vs. 78.3%), greater patient satisfaction, and reduced treatment duration (14.2 vs. 18.7 weeks). Cost-effectiveness improved by 15.8% despite higher coordination costs. Complication rates were lower (8.3% vs. 16.7%) in the interdisciplinary group. Findings support integrated, multi-specialty collaboration for superior dental care outcomes.

## Background:

Contemporary dental practice increasingly encounters patients presenting with complex oral health conditions that transcend the boundaries of individual dental specialties [1]. These multifaceted cases often involve simultaneous periodontal disease, endodontic pathology, orthodontic malocclusion, and prosthodontic rehabilitation needs, necessitating a comprehensive treatment approach that integrates expertise from multiple disciplines [2, 3]. The traditional model of sequential specialty referrals, while historically prevalent, has demonstrated limitations in addressing the interconnected nature of complex dental pathology and may result in fragmented care delivery in many of these situations, these factors may act as barriers to access of ongoing dental care. Additionally, acute oral assessments and timely dental treatment are often required prior to significant medical interventions [4]. The emergence of interdisciplinary dental care represents a paradigm shift toward collaborative treatment planning and execution, where a specialist from various disciplines work together to develop unified treatment strategies [5, 6]. This approach recognizes that optimal outcomes in complex cases require synchronized interventions that consider the biological, functional, and aesthetic aspects of comprehensive oral rehabilitation [7]. Recent evidence suggests that interdisciplinary collaboration significantly improves diagnostic accuracy, treatment predictability, and patient satisfaction compared to traditional approaches [8, 9]. Team-based dental care delivery models have gained considerable attention in recent literature, demonstrating improved efficiency and patient outcomes [10, 11]. Studies have shown that collaborative care approaches yield significant benefits in terms of access to care, particularly among underserved populations, while enhancing treatment effectiveness and healthcare outcomes [12]. The integration of dental hygienists, dental therapists, and various specialists as key members of the dental team has been associated with improved workflow efficiency and reduced procedural errors [13, 14]. Digital technologies have further revolutionized interdisciplinary treatment planning, enabling enhanced communication among team members and improved treatment predictability [15, 16]. Computer-aided design and manufacturing (CAD/CAM) systems, three-dimensional imaging, and digital smile design protocols facilitate collaborative decision-making and allow for more precise treatment execution [17, 18]. These technological advances have made interdisciplinary approaches more feasible and accessible in modern dental practice [19]. Even after promising developments in integrative dental care, significant gaps remain in the understanding of the required implementation strategies and quantitative outcomes of interdisciplinary strategies. Previous research has inadequately compared the effectiveness of interdisciplinary management versus traditional care in terms of clinical outcomes, patient satisfaction, and cost-effectiveness [20, 21]. Furthermore, there are many challenges for successful interdisciplinary collaboration, including communication challenges, role ambiguity, and coordination difficulties, require systematic investigation [22, 23]. Therefore, it is of interest to address these gaps and seeks to compare interdisciplinary versus conventional single-specialty approaches in managing complex dental problems.

## Materials and Methods:

A total of 240 adult patients (aged 18-65 years) presenting with complex dental conditions requiring treatment by at least three different dental specialties was recruited for this study. Complex dental conditions were defined as cases involving simultaneous pathology requiring intervention from periodontics, prosthodontics, endodontics, orthodontics, or oral surgery. Sample size calculation was based on an anticipated 15% difference in treatment success rates between groups, with 80% power and 5% significance level.

## Inclusion criteria:

Patients requiring multi-specialty dental treatment, good general health status (ASA I-II), ability to attended regular follow-up appointments, and willingness to participate in the study duration.

## Exclusion criteria:

Patients with severe systemic diseases affecting treatment outcomes, psychiatric conditions preventing compliance, active substance abuse, pregnancy, or inability to provide inform consent.

## Randomization and group assignment:

Patients were randomly assigned using computer-generated randomization to either the interdisciplinary treatment group (ITG, n=120) or the conventional sequential treatment group (CTG, n=120). Randomization was stratified by age group and case complexity to ensure balanced distribution. Treatment Protocols.

## Interdisciplinary treatment group (ITG):

Patients received coordinated care through weekly interdisciplinary team meetings involving all relevant specialists. Treatment plans were developed collaboratively, with synchronized interventions and continuous communication among team members. Digital treatment planning tools were utilized for case visualization and coordination.

## Conventional treatment group (CTG):

Patients received traditional sequential specialty referrals, with each specialist working independently. Treatment planning occurred within individual specialties without formal coordination mechanisms.

## Equipment and Materials:

Digital radiographic systems (Planmeca ProMax 3D), intraoral scanners (3Shape TRIOS), CAD/CAM systems (CEREC Omnicam), and digital smile design software (DSD Planning Center) were utilized for diagnostic and treatment planning purposes. Standardized clinical protocols were established for both treatment groups to minimize procedural variations.

## Outcome measures:

Primary outcomes included treatment success rates, defined as the achievement of predetermined clinical objectives without major complications. Secondary outcomes encompassed patient satisfaction scores (using validated 10-point Likert scales), total treatment duration, number of appointments required, and comprehensive cost analysis, including direct and indirect expenses.

## Statistical methods:

Data analysis was performed using SPSS version 28.0. Descriptive statistics were calculated for all variables. Independent t-tests were used for continuous variables, while chi-square tests were applied for categorical variables. Analysis of variance (ANOVA) was employed for multiple group comparisons. Statistical significance was set at p<0.05. Intention-to-treat analysis was performed for all primary outcomes.

## Results:

The study successfully enrolled 240 participants, with equal distribution between treatment groups (n=120 each). Mean age was 42.3 ± 12.7 years in the ITG and 43.8 ± 11.9 years in the CTG (p=0.312). Gender distribution showed 58.3% female participants in ITG and 54.2% in CTG (p=0.517). No significant differences were observed in baseline demographic characteristics between groups. The interdisciplinary treatment group demonstrated significantly superior treatment success rates compared to the conventional group. Overall success rates were 92.5 ± 4.2% in ITG versus 78.3 ± 6.8% in CTG (p<0.001). When analyzed by specialty involvement, periodontal treatment success was 94.7% vs. 81.2% (p<0.001), endodontic success was 96.8% vs. 85.4% (p<0.001), and prosthodontic success was 89.3% vs. 74.6% (p<0.001) for ITG and CTG, respectively. Patient satisfaction scores revealed marked differences between treatment approaches. The ITG achieved mean satisfaction scores of 8.7 ± 1.1 compared to 7.2 ± 1.4 in CTG (p<0.001). Specific satisfaction domains showed consistent superiority in the interdisciplinary group: communication quality (9.1 ± 0.8 vs. 7.8 ± 1.2, p<0.001), treatment coordination (8.9 ± 1.0 vs. 6.9 ± 1.5, p<0.001), and overall experience (8.8 ± 1.1 vs. 7.4 ± 1.3, p<0.001). Significant improvements in treatment efficiency were observed in the interdisciplinary approach. Mean treatment duration was reduced from 18.7 ± 5.2 weeks in CTG to 14.2 ± 3.8 weeks in ITG, representing a 23.6% reduction (p<0.001). The number of appointments required was lower in ITG (12.4 ± 2.8 vs. 16.2 ± 4.1, p<0.001). Time to treatment completion showed similar improvements, with ITG patients completing treatment 4.5 weeks earlier on average. Complication rates were significantly lower in the interdisciplinary group. Minor complications occurred in 8.3% of ITG patients compared to 16.7% in CTG (p=0.041). Major complications requiring additional intervention were observed in 2.5% of ITG patients versus 6.7% in CTG (p=0.148). Treatment revisions were necessary in 5.8% of ITG cases compared to 12.5% in CTG (p=0.073). Comprehensive cost analysis revealed favourable economic outcomes for the interdisciplinary approach. Despite higher initial coordination costs, total treatment expenses were reduced by 15.8% in ITG ($4,236 ± $892) compared to CTG ($5,031 ± $1,147) (p=0.007). Decreased treatment duration, fewer complications, and reduced revision procedures were decisive factors in this reduction of expenses. Cost per successful treatment outcome was $4,580 in ITG versus $6,430 in CTG. Post-treatment quality of life assessments using the Oral Health Impact Profile showed significant improvements in both groups, with greater benefits observed in ITG. Functional improvement scores were 7.8 ± 1.3 in ITG versus 6.9 ± 1.6 in CTG (p=0.001). Aesthetic satisfaction scores were 8.2 ± 1.2 in ITG compared to 7.1 ± 1.5 in CTG (p<0.001).

## Discussion:

The study found strong evidence proving the superiority of interdisciplinary approaches in managing complex dental and oral health conditions. 14.2% improvement in treatment success rates found by the present study demonstrates the clinical advantage of coordinated specialty care over traditional approaches. These results can be compared with previous research which also highlighted the benefits of collaborative dental care delivery models [12, 13]. Better patient satisfaction outcomes observed in the interdisciplinary group further confirmed that communication and care coordination also improved simultaneously which was consistent with findings from previous team-based healthcare delivery studies [14, 15]. The significant improvement in satisfaction scores (8.7 vs. 7.2) suggests that patients value the coordinated approach and perceive higher quality care when specialists work collaboratively. This finding supports the patient-centered care model advocated in contemporary healthcare literature [16]. The 23.6% reduction in treatment duration represents a substantial improvement in healthcare efficiency. This finding contradicts common assumptions that interdisciplinary approaches might be more time-consuming due to coordination requirements [17]. Instead, our results suggest that early collaborative planning and synchronized interventions accelerate treatment completion. Similar efficiency improvements have been reported in medical team-based care models [18, 19]. Cost-effectiveness analysis revealed unexpected economic benefits of the interdisciplinary approach, despite higher initial coordination costs. The 15.8% reduction in total treatment costs primarily resulted from decreased complication rates and reduced need for treatment revisions [20]. These findings have important implications for healthcare policy and insurance coverage decisions, suggesting that investment in collaborative care infrastructure may yield long-term cost savings [21]. The digital technology integration observed in successful interdisciplinary cases supports recent literature emphasizing the role of technology in facilitating collaborative care [22]. Digital treatment planning tools, 3D imaging, and CAD/CAM systems enhanced communication among specialists and improved treatment predictability [23]. The adoption of digital workflows appears essential for effective interdisciplinary practice in modern dentistry. Despite the positive outcomes, several barriers to interdisciplinary implementation were identified during the study. Communication challenges, scheduling coordination difficulties, and role ambiguity occasionally impacted treatment delivery. These findings are consistent with barriers reported in healthcare team literature and suggest areas for future improvement. Addressing these challenges through structured communication protocols and clear role definitions may further enhance interdisciplinary effectiveness. The lower complication rates observed in the interdisciplinary group may result from comprehensive treatment planning that considers interactions between different treatment modalities. When specialists collaborate from the outset, potential conflicts and complications can be anticipated and prevented. This proactive approach contrasts with reactive problem-solving often required in sequential treatment models.

## Study strengths and limitations:

This study's strengths include its prospective randomized design, comprehensive outcome assessment, and substantial sample size. The inclusion of cost-effectiveness analysis provides valuable economic data for healthcare decision-making. The use of validated patient satisfaction instruments enhances the reliability of subjective outcome measures. However, several limitations must be acknowledged. The study was conducted at a single academic institution, which may limit generalizability to private practice settings. The relatively short follow-up period may not capture long-term treatment stability. Additionally, the learning curve associated with implementing interdisciplinary protocols may have influenced early outcomes. Future multi-center studies with longer follow-up periods would strengthen the evidence base for interdisciplinary care.

## Conclusion:

This study confirms that interdisciplinary dental care improves clinical outcomes, patient satisfaction, efficiency, and cost-effectiveness over conventional approaches. Digital technologies play a key role in enabling effective collaboration. Future efforts should refine protocols, overcome barriers, and assess the long-term benefits of team-based care.
